# Author Correction: A comprehensive map coupling histone modifications with gene regulation in adult dopaminergic and serotonergic neurons

**DOI:** 10.1038/s41467-018-07154-5

**Published:** 2018-11-02

**Authors:** Erik Södersten, Konstantinos Toskas, Vilma Rraklli, Katarina Tiklova, Åsa K. Björklund, Markus Ringnér, Thomas Perlmann, Johan Holmberg

**Affiliations:** 1 Department of Cell and Molecular Biology, Ludwig Institute for Cancer Research, Karolinska Institutet, Nobels väg 3, 171 77 Stockholm, Sweden; 2 Department for Cell and Molecular Biology, National Bioinformatics Infrastructure Sweden, Science for Life Laboratory, Uppsala University, 751 24 Uppsala, Sweden; 3 Department of Biology, National Bioinformatics Infrastructure Sweden, Science for Life Laboratory, Lund University, 223 62 Lund, Sweden

Correction to: *Nature Communications*; 10.1038/s41467-018-03538-9; published online 26 March 2018

In the originally published version of this Article, financial support was not fully acknowledged. The PDF and HTML versions of the Article have now been corrected to include support to Thomas Perlmann provided by Knut and Alice Wallenberg Foundation (grant 2013.0075) and Swedish Research Council (VR; grant 2016-02506).

