# Supplementary material for: A comprehensive map coupling histone modifications with gene regulation in adult dopaminergic and serotonergic neurons
Source: Nat Commun. 2018 Mar 26;9:1226. doi: 10.1038/s41467-018-03538-9 (PMC5964330; doi:10.1038/s41467-018-03538-9)
Supplement: Supplementary file 3 — Description of Additional Supplementary Files [file 41467_2018_3538_MOESM3_ESM.pdf]

Description of Additional Supplementary Files

File Name: Supplementary Data 1
Description: Genes with significantly higher expression levels in mDA-neurons vs NPC and associated chromatin states.

File Name: Supplementary Data 2
Description: Genes with significantly lower expression levels in mDA-neurons vs NPC and associated chromatin states.

File Name: Supplementary Data 3
Description: Genes with expression levels: $\log2(RPKM+1)<1$ in mDA-neurons and NPC, determined as “silent”.

File Name: Supplementary Data 4
Description: Differentially expressed genes between mDA-neurons and SER-neurons.

File Name: Supplementary Data 5
Description: Differentially expressed genes after 6-OHDA and methamphetamine treatment, detected by TRAP-seq.

File Name: Supplementary Data 6
Description: Total chromatin states and associated RPKMs for all genes in all samples of mDA-neurons, SER-neurons and NPCs.
