# Supplementary material for: A comprehensive map coupling histone modifications with gene regulation in adult dopaminergic and serotonergic neurons
Source: Nat Commun. 2018 Mar 26;9:1226. doi: 10.1038/s41467-018-03538-9 (PMC5964330; doi:10.1038/s41467-018-03538-9)
Supplement: Supplementary file 1 — Supplementary Information [file 41467_2018_3538_MOESM1_ESM.pdf]

**Supplementary Information**

**Title:** A comprehensive map coupling histone modifications with gene regulation in adult dopaminergic and serotonergic neurons

Södersten et. al.

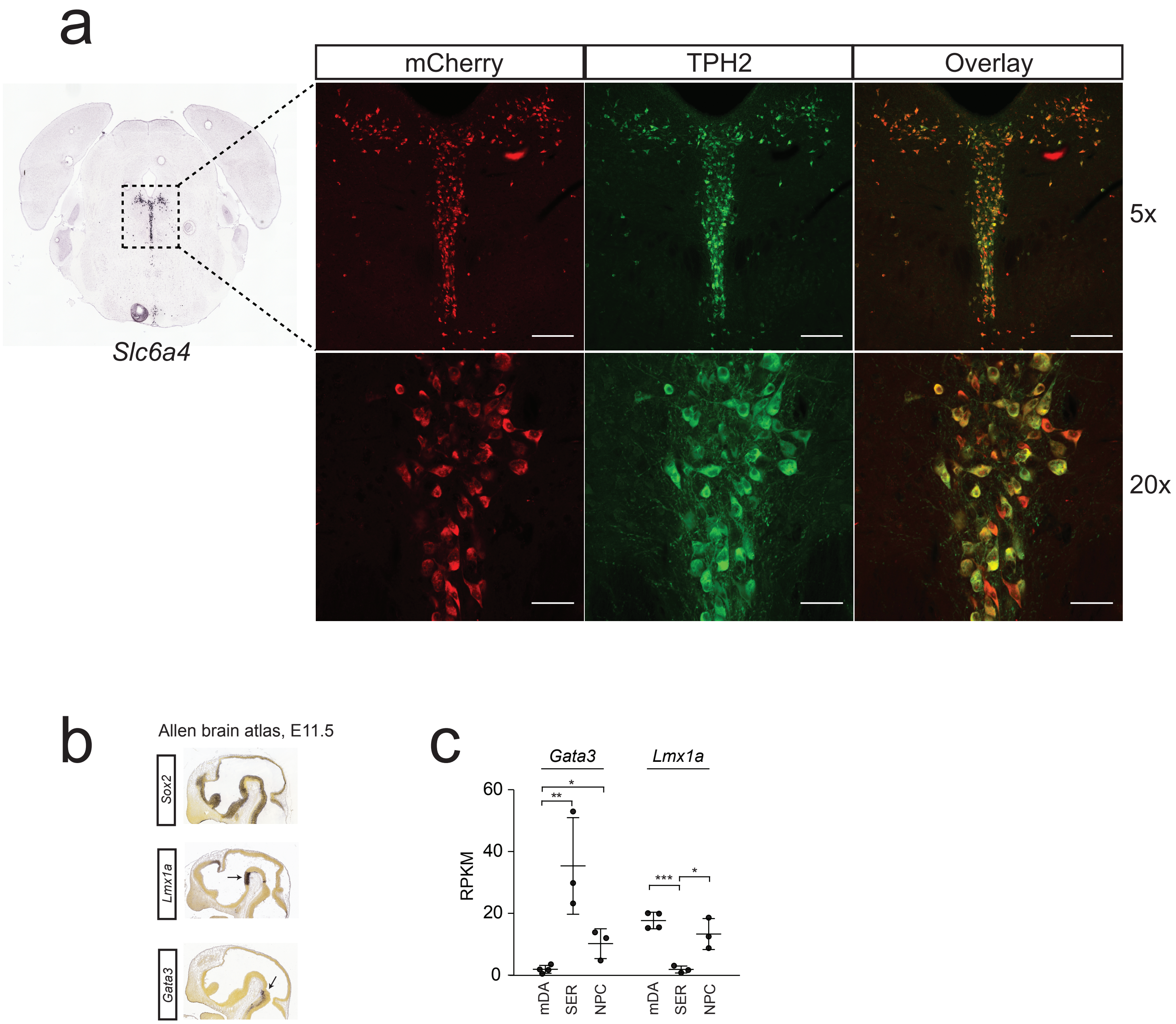
a
mCherry
TPH2
Overlay
5x
20x
Slc6a4
b
Allen brain atlas, E11.5
Sox2
Lmx1a
Gata3
c
Gata3
Lmx1a
60
40
20
0
RPKM
**
*
***
*
mDA
SER
NPC
mDA
SER
NPC

**Supplementary Figure 1**

**a)** Rpl10A-mCherry expression (red) overlap TPH2 expression (green) in the hindbrain in *SERT-Cre-Rpl10a-mCherry* mice. 5x and 20x magnification. To the left is an *in situ* hybridization for *Slc6a4* taken from the Allen Brain Atlas (mouse.brain-map.org) indicating the position of the images. Scale bars: 200 µm (upper panels), 50 µm (lower panels). **b)** *In situ* hybridizations in E11.5 embryos for *Sox2*, *Lmx1a* and *Gata3* taken from Allen Brain Atlas showing expression of midbrain dopaminergic (*Lmx1a*) and raphe serotonergic progenitor (*Gata3*) marker genes within the *Sox2*-positive population. **c)** Bars indicate average RPKM ± SD for the serotonergic progenitor marker *Gata3* and *Lmx1a* in mDA (n = 4), SER (n = 3) and NPC (n = 3) RNA-seq libraries. Significance according to two-tailed Student´s t-test assuming equal variances. *** $p \leq 0.001$, ** $p \leq 0.01$, * $p \leq 0.05$.

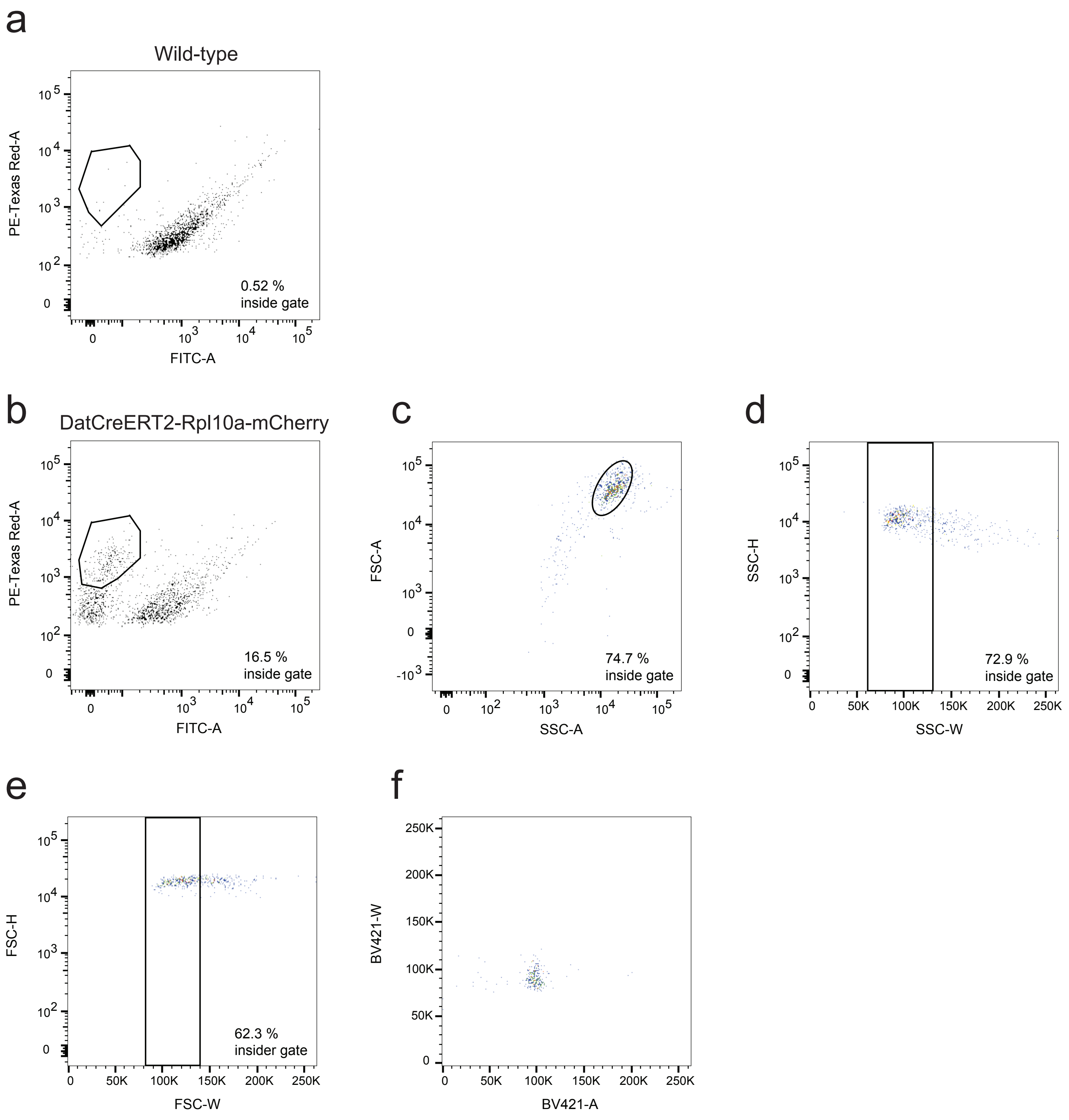
a
Wild-type
PE-Texas Red-A
FITC-A
0.52 %
inside gate
b
DatCreERT2-Rpl10a-mCherry
PE-Texas Red-A
FITC-A
16.5 %
inside gate
c
FSC-A
SSC-A
74.7 %
inside gate
d
SSC-H
SSC-W
72.9 %
inside gate
e
FSC-H
FSC-W
62.3 %
insider gate
f
BV421-W
BV421-A

**Supplementary Figure 2**

FACS strategy for sorting single mDA-nuclei from *DatCreERT2-Rpl10a-mCherry* mice. **a)** Midbrain nuclei from *DatCreERT2*-/- *Rpl10a-mCherry*+/- mice (wild-type) did not contain a population that appeared in **b)** midbrain nuclei from *DatCreERT2*+/+ *Rpl10a-mCherry*+/- mice. **c-e)** Further gating for forward-scattered and side-scattered light for selecting events of correct size. **f)** Addition of DAPI suggested a majority of single nuclei. FACS statistics: Gate 1 (PE-Texas Red A, FITC-A) 16.5% of parent. Gate 2 (FSC-A, SSC-A) 74.7% of parent. Gate 3 (SSC-H, SSC-W) 72.9% of parent. Gate 4 (FSC-H, FSC-W) 62.3% of parent.

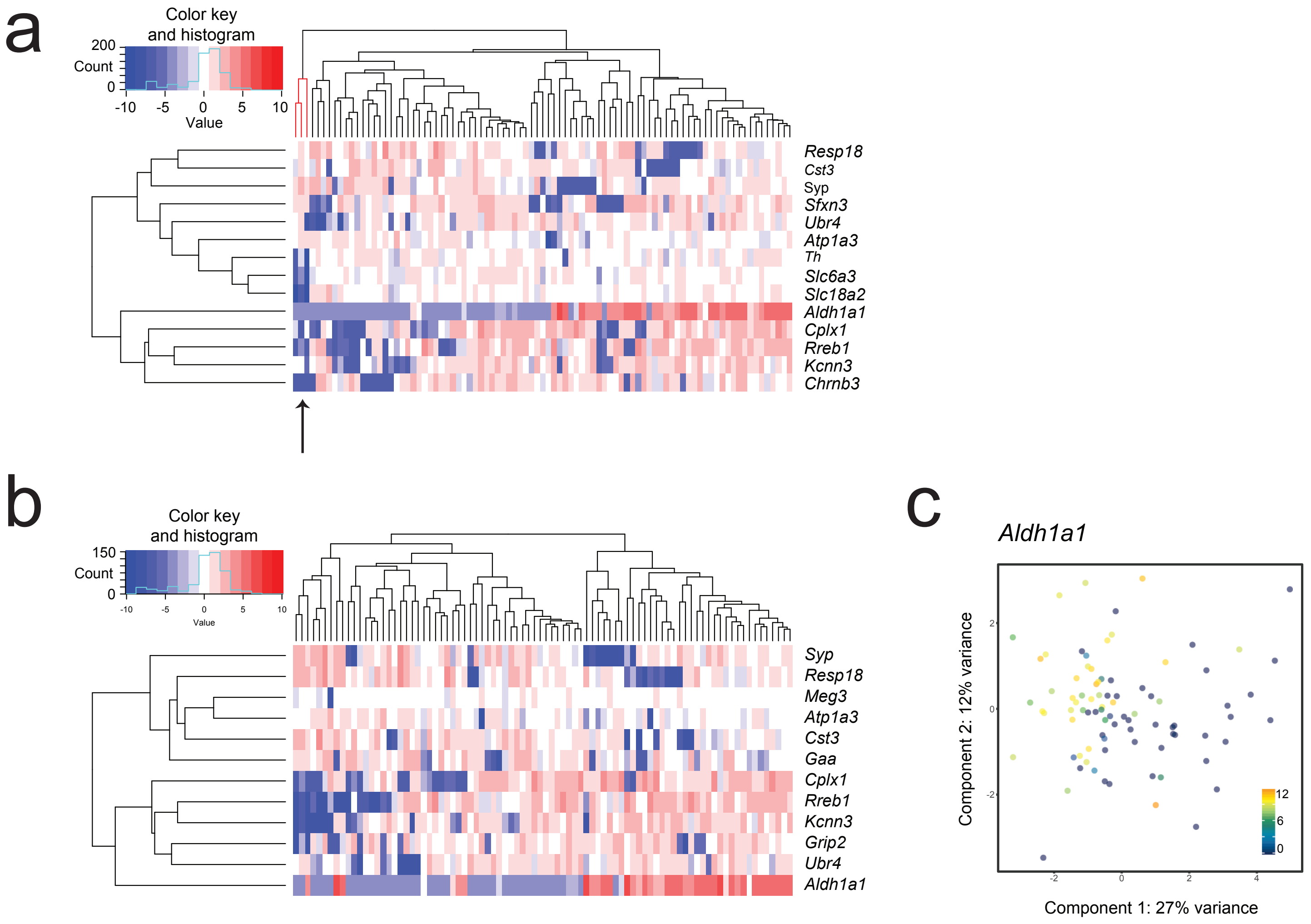
a
Color key
and histogram
200
Count
0
-10
-5
0
5
10
Value
Resp18
Cst3
Syp
Sfxn3
Ubr4
Atp1a3
Th
Slc6a3
Slc18a2
Aldh1a1
Cplx1
Rreb1
Kcnn3
Chrnb3
b
Color key
and histogram
150
Count
0
Syp
Resp18
Meg3
Atp1a3
Cst3
Gaa
Cplx1
Rreb1
Kcnn3
Grip2
Ubr4
Aldh1a1
c
Aldh1a1
Component 2: 12% variance
Component 1: 27% variance
12
6
0

**Supplementary Figure 3**

Single-nuclei RNA-seq. **a)** Heatmap of mean-centred normalized log-expression values for correlated HVGs in mCherry positive nuclei (n = 89). Dendrograms are formed by hierarchical clustering on the Euclidean distances between genes (rows) or cells (columns). Column color black and red indicate the cluster to which each cell is assigned after a dynamic tree cut. The arrow indicates a cluster of 3 cells displaying low expression of dopamine neuron markers. **b)** Heatmap of mean-centred normalized log-expression values for correlated HVGs in 86 nuclei after removal of three nuclei from non-mDA cells. Two subpopulations of mDA-neurons were detected by hierarchical clustering on the Euclidean distances between genes (row) or cells (column). **c)** PCA plot constructed from normalized log-expression values of correlated HVGs after removal of 3 nuclei from non-mDA cells. Each nucleus is colored according to the expression of *Aldh1a1*, which also is a determinant for the two subpopulations detected in (**b**).

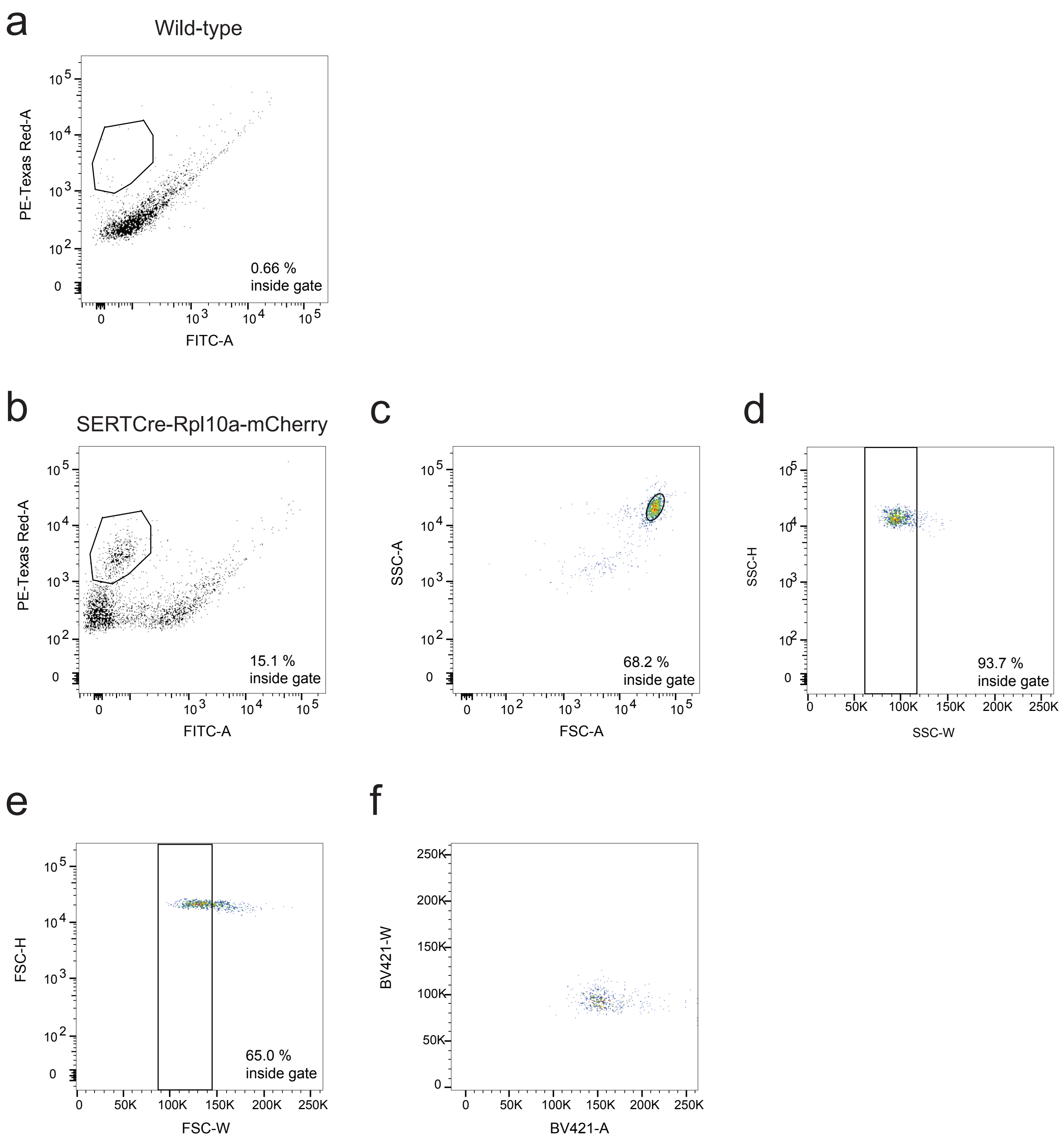
a
Wild-type
PE-Texas Red-A
FITC-A
0.66 %
inside gate
b
SERTCre-Rpl10a-mCherry
PE-Texas Red-A
FITC-A
15.1 %
inside gate
c
SSC-A
FSC-A
68.2 %
inside gate
d
SSC-H
SSC-W
93.7 %
inside gate
e
FSC-H
FSC-W
65.0 %
inside gate
f
BV421-W
BV421-A

**Supplementary Figure 4**

FACS strategy for sorting single SER-nuclei from *SERT-Cre-Rpl10a-mCherry* mice. **a)** Hindbrain nuclei from *SERT-Cre* -/- *Rpl10a-mCherry*+/- mice (wild-type) did not display a population that appeared in **b)** hindbrain nuclei from *SERT-Cre* +/+ *Rpl10a-mCherry*+/- mice. **c-e)** Further gating for forward-scattered and side-scattered light for selecting events of correct size. **f)** Addition of DAPI suggested a majority of single nuclei. FACS statistics: Gate 1 (PE-Texas Red A, FITC-A) 15.1% of parent. Gate 2 (FSC-A, SSC-A) 68.2% of parent. Gate 3 (SSC-H, SSC-W) 94.7% of parent. Gate 4 (FSC-H, FSC-W) 65.0% of parent.

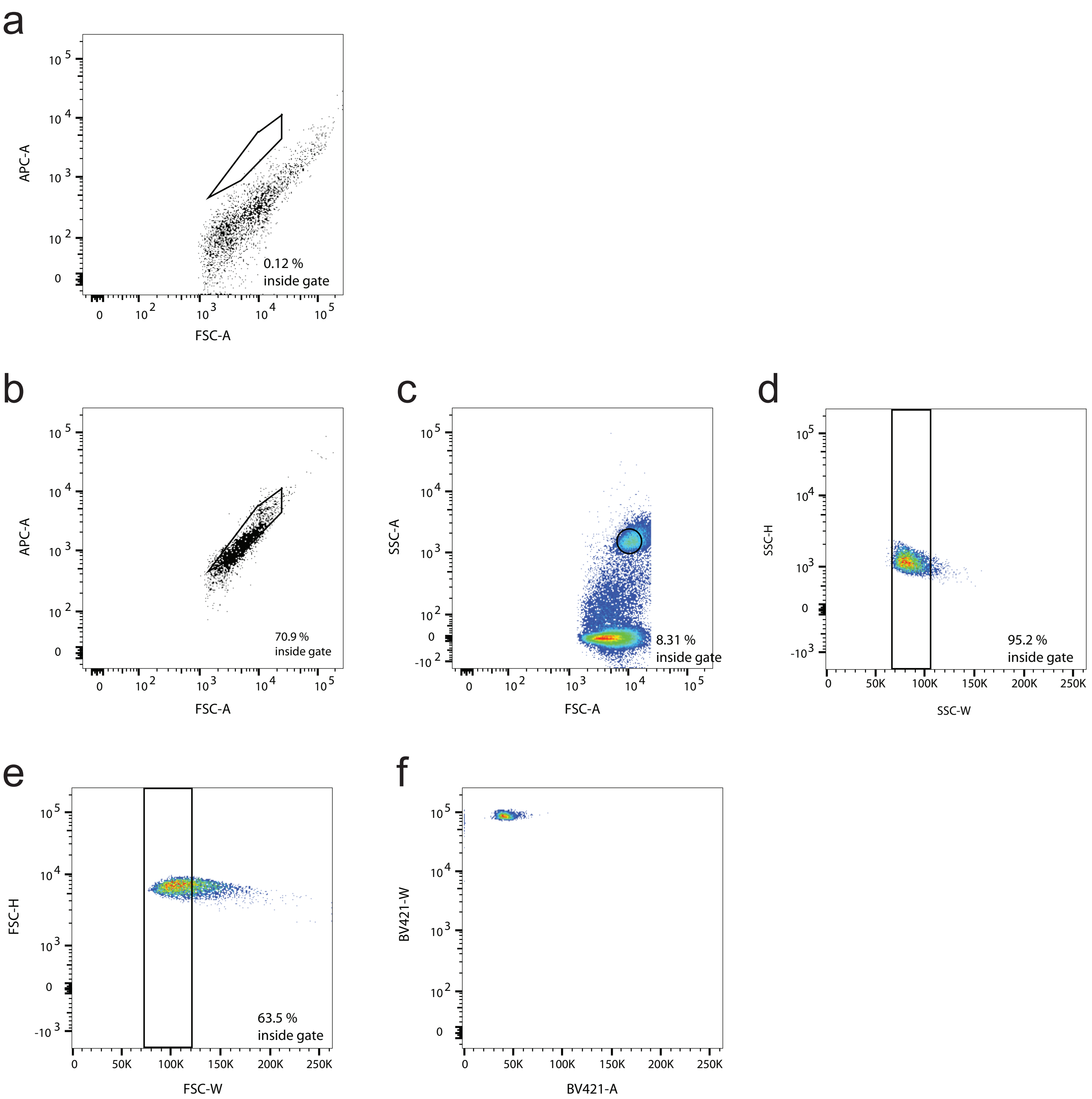
a
APC-A
FSC-A
0.12 %
inside gate
b
APC-A
FSC-A
70.9 %
inside gate
c
SSC-A
FSC-A
8.31 %
inside gate
d
SSC-H
SSC-W
95.2 %
inside gate
e
FSC-H
FSC-W
63.5 %
inside gate
f
BV421-W
BV421-A
0
50K
100K
150K
200K
250K

**Supplementary Figure 5**

FACS strategy for sorting single NPC-nuclei from E11.5 embryonic brain. **a)** Unstained E11.5 nuclei did not display a population of events that appeared in **b)** E11.5 nuclei after incubation with a Alexa 647 conjugated anti-SOX2 antibody. **c-e)** Further gating for forward-scattered and side-scattered light for selecting events of correct size. **f)** Addition of DAPI suggested a majority of single nuclei. FACS statistics: Gate 1 (APC-A, FSC-A) 70.9% of parent. Gate 2 (SSC-A, FSC-A) 8.31% of parent. Gate 3 (SSC-H, SSC-W) 95.2% of parent. Gate 4 (FSC-H, FSC-W) 63.5% of parent.

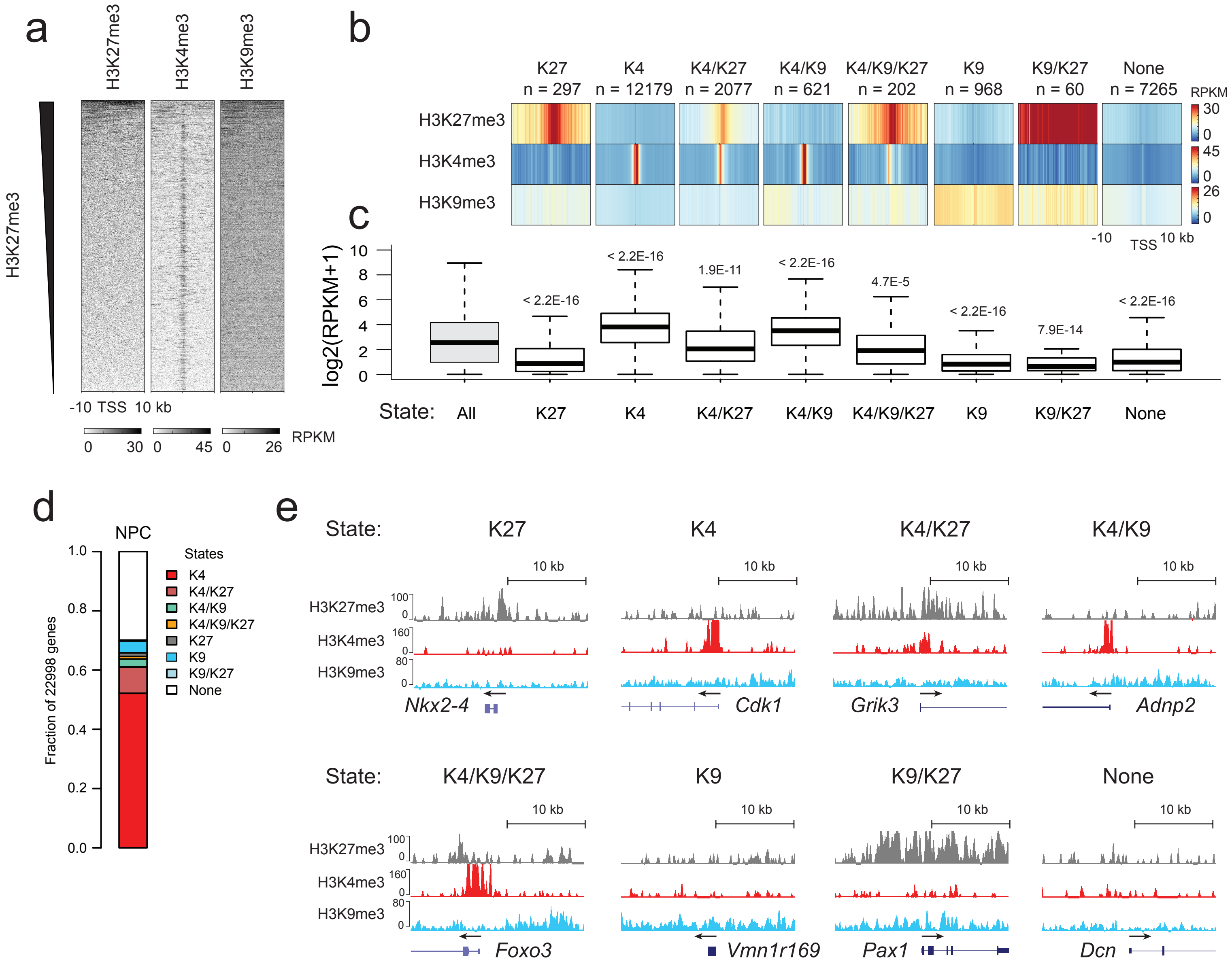

a
H3K27me3
H3K4me3
H3K9me3
H3K27me3
-10 TSS 10 kb
0 30 0 45 0 26 RPKM
b
K27 n = 297
K4 n = 12179
K4/K27 n = 2077
K4/K9 n = 621
K4/K9/K27 n = 202
K9 n = 968
K9/K27 n = 60
None n = 7265
H3K27me3
H3K4me3
H3K9me3
RPKM 30 0 45 0 26 0
-10 TSS 10 kb
c
log2(RPKM+1)
< 2.2E-16
< 2.2E-16
1.9E-11
< 2.2E-16
4.7E-5
< 2.2E-16
7.9E-14
< 2.2E-16
State: All K27 K4 K4/K27 K4/K9 K4/K9/K27 K9 K9/K27 None
d
NPC
Fraction of 22998 genes
States
K4
K4/K27
K4/K9
K4/K9/K27
K27
K9
K9/K27
None
e
State: K27 K4 K4/K27 K4/K9
10 kb
H3K27me3
H3K4me3
H3K9me3
Nkx2-4
Cdk1
Grik3
Adnp2
State: K4/K9/K27 K9 K9/K27 None
Foxo3
Vmn1r169
Pax1
Dcn

**Supplementary Figure 6**

Validation of ChIP-seq in E11.5 NPCs. **a)** H3K27me3 and H3K9me3 abundance around TSS is inversely correlated with H3K4me3 abundance in NPC: Heatmaps showing the genome-wide abundance of H3K27me3, H3K4me3 and H3K9me3 ± 10 kb around TSS of individual genes in NPC as RPKMs obtained by ChIP-seq. Heatmaps were sorted for descending H3K27me3 abundance. **b)** Categorization of TSS regions into chromatin states: Heatmap profiles of H3K27me3, H3K4me3 and H3K9me3 RPKMs ± 10 kb around TSS of genes per defined chromatin state in NPC (denoted as K27, K4, K4/K27, K4/K9, K4/K9/K27, K9, K9/K27 and None, respectively). **c)** Individual chromatin states are associated with different levels of gene expression in NPC: box-plots showing the expression levels (log2(RPKM+1)) of genes in the different chromatin-state categories. Centre line is median, bounds are 25th and 75th percentiles and whiskers are ±1.5 IQR. Chromatin states with an average gene expression that is different compared to the global average gene expression are indicated by p-values obtained by a two-sided Wilcoxon rank sum test. **d)** Genome-wide relative abundance of ChIP-seq defined chromatin states ± 10 kb around TSS in NPC. **e)** UCSC Genome Browser excerpts (https://genome.ucsc.edu) showing examples of enrichment for H3K27me3 (gray, vertical scale 0-100 RPKM), H3K4me3 (red, vertical scale 0-160 RPKM) and H3K9me3 (blue, vertical scale 0-80 RPKM) at representative genes per chromatin state in NPC. Each plot spans 20 kb.

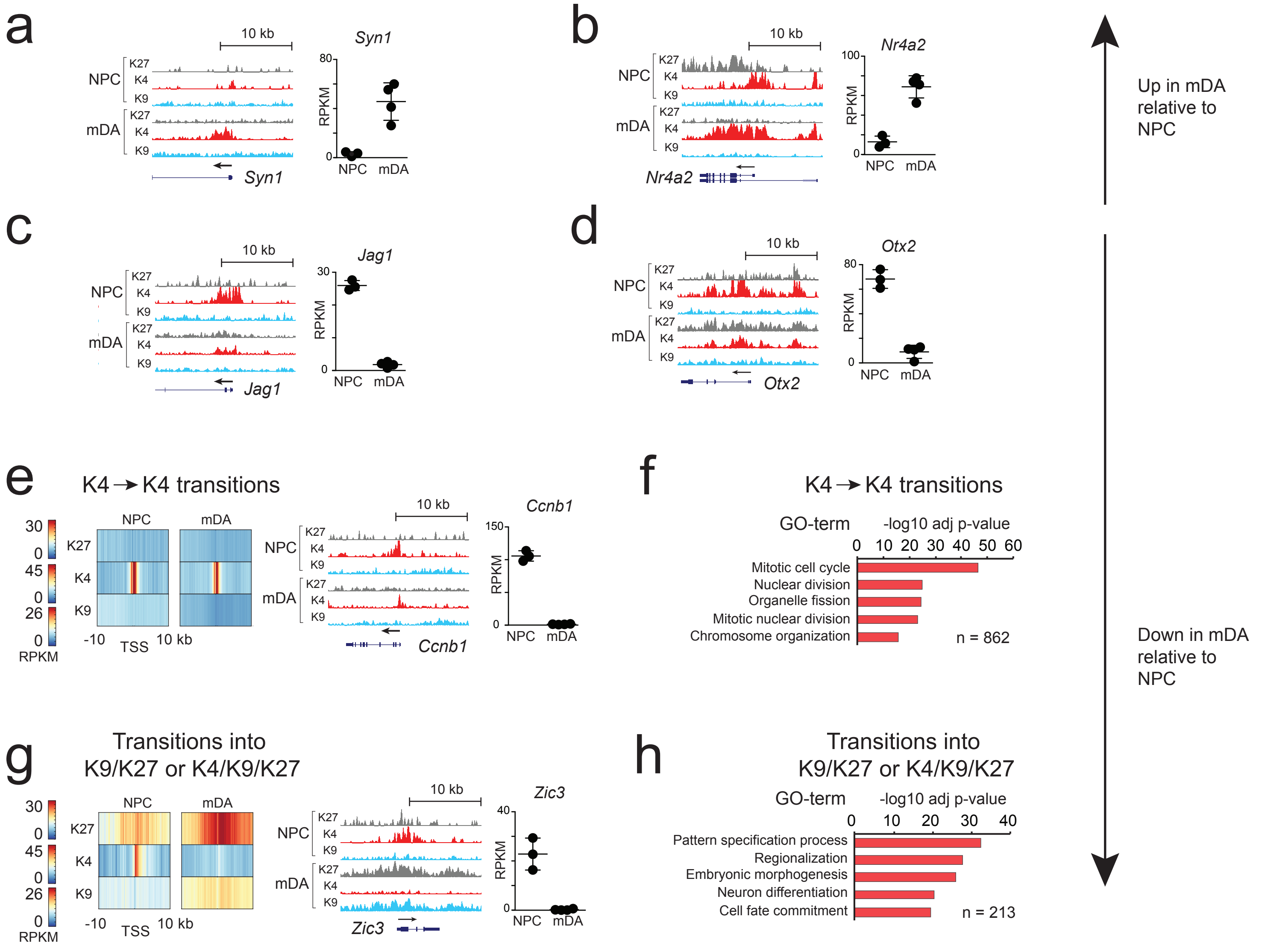


i

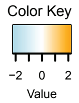


log2(RPKM+1)<1 in NPC and mDA

| State in mDA \ State in NPC | K4 | K4/K27 | K4/K9 | K4/K9/K27 | K27 | K9 | K9/K27 | None |
|---|---|---|---|---|---|---|---|---|
| K4 | 500 | 55 | 19 | 7 | 7 | 16 | 0 | 193 |
| K4/K27 | 147 | 152 | 2 | 8 | 31 | 2 | 1 | 105 |
| K4/K9 | 10 | 2 | 8 | 1 | 1 | 14 | 0 | 22 |
| K4/K9/K27 | 14 | 56 | 6 | 19 | 26 | 9 | 17 | 30 |
| K27 | 30 | 30 | 1 | 2 | 18 | 12 | 0 | 246 |
| K9 | 1 | 0 | 3 | 0 | 0 | 227 | 0 | 264 |
| K9/K27 | 12 | 43 | 0 | 12 | 34 | 73 | 17 | 123 |
| None | 65 | 0 | 3 | 0 | 17 | 134 | 0 | 2227 |

**Supplementary Figure 7**

**a-d)** *Left:* UCSC Genome Browser excerpts showing examples of enrichment for H3K27me3 (vertical scale 0-100 RPKM), H3K4me3 (vertical scale 0-160 RPKM) and H3K9me3 (vertical scale 0-80 RPKM) at representative genes per transition. The plots span 20 kb. *Right:* Bars indicate average RPKM ± SD. for representative genes per chromatin state transition. *Syn1* (**a**) exemplifies K4 to K4 transitions for an up-regulated gene from NPC to mDA, (**b**) *Nr4a2* exemplifies K4/K27 to K4 transition for a up-regulated gene from NPC to mDA. (**c**) *Jag1* exemplifies K4 to K4/K27 transitions for a down-regulated gene from NPC to mDA and (**d**) *Otx2* exemplifies K4/K27 to K4/K27 transitions for a down-regulated gene from NPC to mDA. Bars in **a-d** indicate average RPKM ± SD. **e)** *Left:* Heatmap profiles of average H3K27me3, H3K4me3 and H3K9me3 RPKMs ± 10 kb around TSS of genes that were down-regulated from NPC to mDA and categorized as K4 in NPC and K4 in mDA. *Middle:* UCSC Genome Browser excerpt as in (**a**) for *Ccnb1*, exemplifying a K4 to K4 transition for a gene down-regulated from NPC to mDA. *Right:* Bars indicate average RPKM ± SD. for *Ccnb1* in NPC and mDA. **f)** Gene Ontology (GO) of top (-log10(adj. p-value), Fisher's exact test) biological processes for down-regulated genes in mDA relative to NPC that were K4 in NPC and K4 in mDA. **g)** *Left:* Heatmap profiles as in (**e**) of genes that were down-regulated from NPC to mDA and categorized as K9/K27 or K4/K9/K27 in mDA. *Middle:* UCSC Genome Browser excerpt as in (**a**) for *Zic3*, exemplifying a transition into K9/K27 for a down-regulated gene from NPC to mDA. *Right:* Bars indicate average RPKM ± s.e.m. for *Zic3* in NPC and mDA. **h)** Gene Ontology (GO) of top (-log10(adj. p-value), Fisher's exact test) biological processes for down-regulated genes in mDA relative to NPC that were K4/K9/K27 and K9/K27 in mDA. **i)** Quantification of chromatin-state transitions between NPC and mDA neurons for genes that were silent (log2(RPKM+1) < 1) in NPC and mDA. Colored squares indicate transitions that occurred significantly more often than expected (odds ratio, orange = more than expected, blue = less than expected, $p<0.05$ according to Fisher's exact test and adjusted for multiple testing).

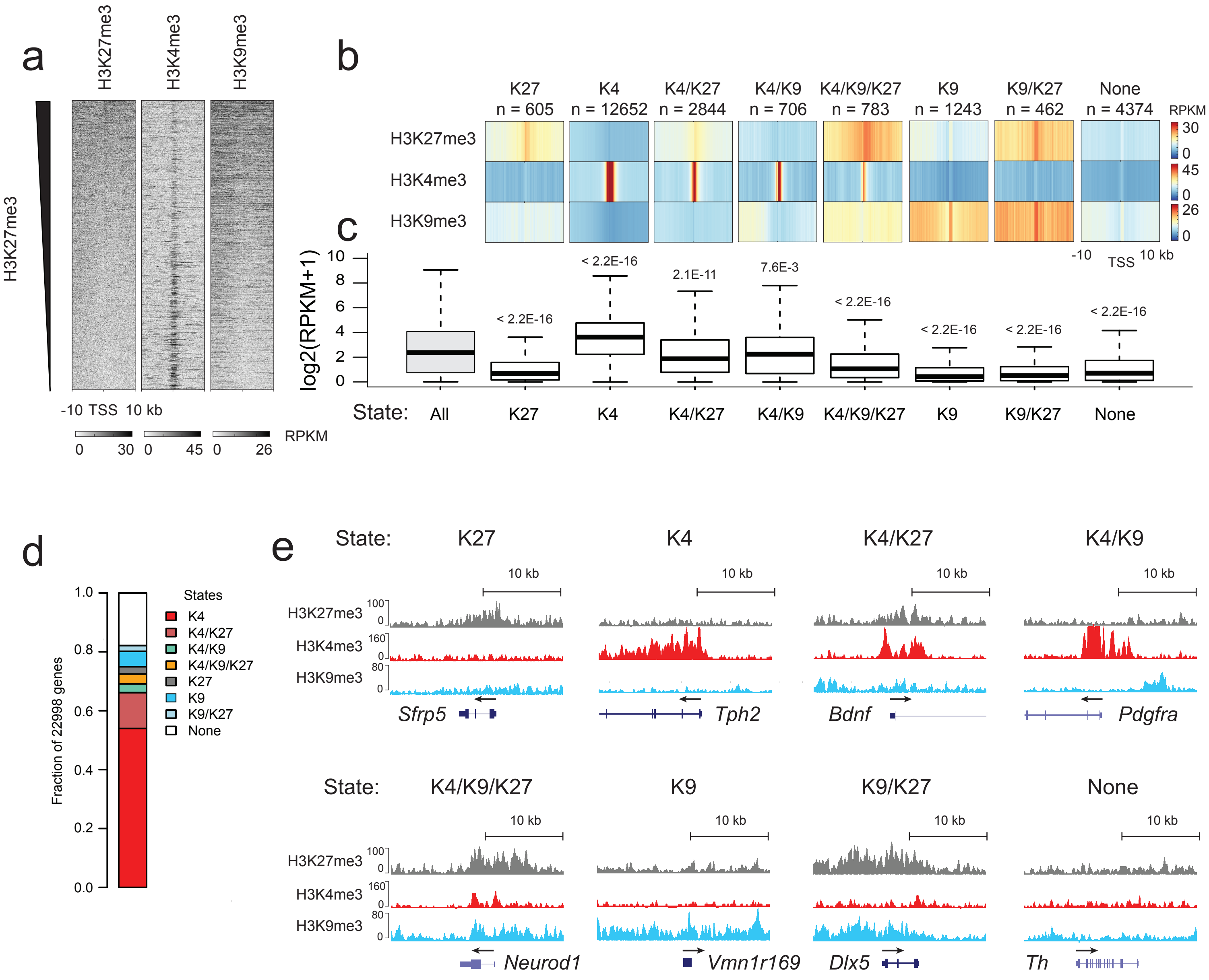
a
H3K27me3
H3K4me3
H3K9me3
-10 TSS 10 kb
0 30 0 45 0 26 RPKM
b
K27 n = 605
K4 n = 12652
K4/K27 n = 2844
K4/K9 n = 706
K4/K9/K27 n = 783
K9 n = 1243
K9/K27 n = 462
None n = 4374
RPKM 30 0 45 0 26 0
-10 TSS 10 kb
c
log2(RPKM+1)
< 2.2E-16
2.1E-11
7.6E-3
State: All K27 K4 K4/K27 K4/K9 K4/K9/K27 K9 K9/K27 None
d
Fraction of 22998 genes
States
K4
K4/K27
K4/K9
K4/K9/K27
K27
K9
K9/K27
None
e
State: K27 K4 K4/K27 K4/K9
10 kb
Sfrp5
Tph2
Bdnf
Pdgfra
State: K4/K9/K27 K9 K9/K27 None
Neurod1
Vmn1r169
Dlx5
Th

**Supplementary Figure 8**

Validation of ChIP-seq in SER-neurons. **a)** H3K27me3 and H3K9me3 abundance around TSS is inversely correlated with H3K4me3 abundance in SER neurons: Heatmaps showing the genome-wide abundance of H3K27me3, H3K4me3 and H3K9me3 ± 10 kb around TSS of individual genes in SER-neurons as RPKMs obtained by ChIP-seq. Heatmaps were sorted for descending H3K27me3 abundance. **b)** Categorization of TSS regions into chromatin states: Heatmap profiles of H3K27me3, H3K4me3 and H3K9me3 RPKMs ± 10 kb around TSS of genes per defined chromatin state in SER-neurons (denoted as K27, K4, K4/K27, K4/K9, K4/K9/K27, K9, K9/K27 and None, respectively). **c)** Individual chromatin states are associated with different levels of gene expression in SER-neurons: box-plots showing the expression levels (log2(RPKM+1)) of genes in the different chromatin-state categories. Centre line is median, bounds are 25th and 75th percentiles and whiskers are ±1.5 IQR. Chromatin states with an average gene expression that is different compared to the global average gene expression are indicated by p-values obtained by a two-sided Wilcoxon rank sum test. **d)** Genome-wide relative abundance of ChIP-seq defined chromatin states ± 10 kb around TSS in SER-neurons. **e)** UCSC Genome Browser excerpts (https://genome.ucsc.edu) showing examples of enrichment for H3K27me3 (gray, vertical scale 0-100 RPKM), H3K4me3 (red, vertical scale 0-160 RPKM) and H3K9me3 (blue, vertical scale 0-80 RPKM) at representative genes per chromatin state in SER-neurons. Each plot spans 20 kb.

S9

a

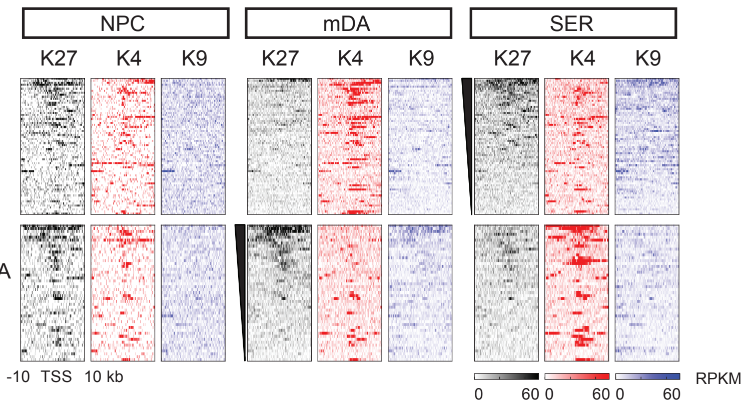


b

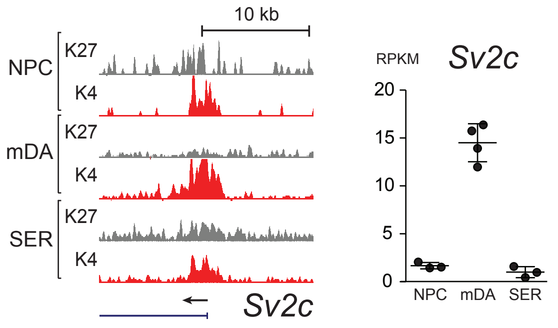


c

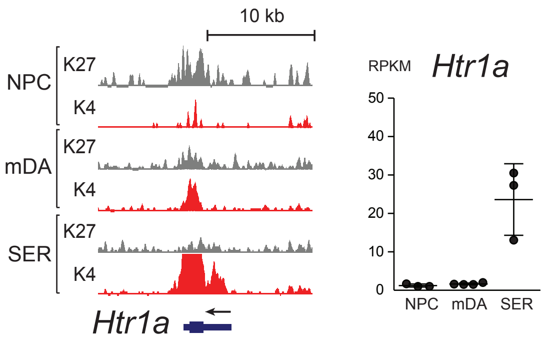


d

Color Key −2 0 2

Up in mDA vs SER

| State in mDA \ State in NPC | K4 | K4/K27 | K4/K9 | K4/K9/K27 | K27 | K9 | K9/K27 | None |
|---|---|---|---|---|---|---|---|---|
| K4 | 99 | 51 | 3 | 4 | 0 | 2 | 0 | 8 |
| K4/K27 | 11 | 13 | 0 | 0 | 0 | 0 | 0 | 3 |
| K4/K9 | 3 | 1 | 2 | 1 | 0 | 0 | 1 | 0 |
| K4/K9/K27 | 0 | 4 | 1 | 0 | 0 | 0 | 0 | 0 |
| K27 | 0 | 0 | 0 | 0 | 0 | 0 | 0 | 4 |
| K9 | 0 | 0 | 0 | 0 | 0 | 3 | 0 | 6 |
| K9/K27 | 0 | 0 | 0 | 0 | 0 | 1 | 0 | 0 |
| None | 1 | 1 | 0 | 0 | 1 | 2 | 0 | 46 |

e

Color Key −2 0 2

Down in mDA vs SER

| State in mDA \ State in NPC | K4 | K4/K27 | K4/K9 | K4/K9/K27 | K27 | K9 | K9/K27 | None |
|---|---|---|---|---|---|---|---|---|
| K4 | 33 | 12 | 1 | 0 | 1 | 0 | 0 | 3 |
| K4/K27 | 21 | 24 | 1 | 1 | 4 | 0 | 0 | 2 |
| K4/K9 | 1 | 0 | 0 | 0 | 0 | 0 | 0 | 0 |
| K4/K9/K27 | 1 | 9 | 1 | 2 | 0 | 1 | 0 | 2 |
| K27 | 6 | 3 | 0 | 0 | 0 | 0 | 0 | 9 |
| K9 | 0 | 0 | 0 | 0 | 0 | 2 | 0 | 0 |
| K9/K27 | 2 | 2 | 0 | 2 | 0 | 0 | 0 | 1 |
| None | 0 | 0 | 0 | 0 | 0 | 0 | 0 | 13 |

f

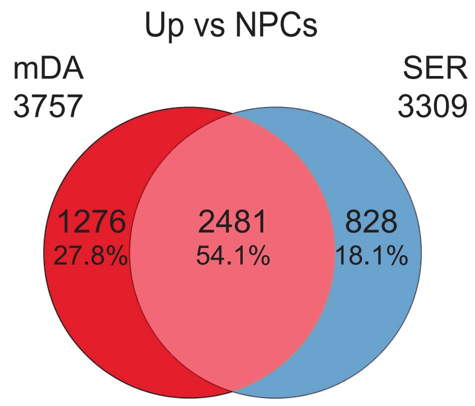


g

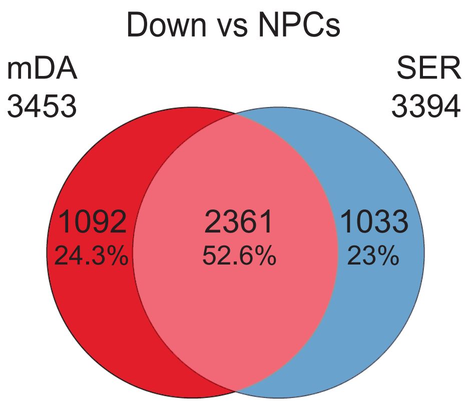

**Supplementary Figure 9**

Repressed genes belonging to the K4/K27 state in NPC are selectively de-repressed in SER or mDA-neurons. **a)** ChIP-seq heatmaps showing abundance of H3K27me3, H3K4me3 and H3K9me3 as RPKMs ± 10 kb around TSS of genes associated with the chromatin state K4/K27 in NPC and that were up-regulated in mDA relative to SER (top panel) and down-regulated in mDA relative to SER (bottom panel). Heatmaps were sorted for descending H3K27me3 abundance as indicated by black gradients. **b-c)** *Left panels:* UCSC Genome Browser excerpt showing examples of enrichment for H3K27me3 (K27, gray, vertical scale 0-100 RPKM) and H3K4me3 (K4, red, vertical scale 0-160 RPKM) around the *Sv2c* and *Htr1a* loci in NPC, mDA and SER. The plots span 20 kb. *Right panels:* Bars indicate average RPKM ± SD in NPC, mDA and SER for *Sv2c* and *Htr1a* respectively. **d)** Quantification of chromatin-states in mDA and NPC for up-regulated genes in mDA relative to SER. Colored squares indicate transitions that occurred significantly more often than expected (odds ratio, orange = more than expected, blue = less than expected, $p < 0.05$ according to Fisher's exact test and adjusted for multiple testing). **e)** As in (b) for down-regulated genes in mDA relative to SER. **f-g)** Comparison of up-regulated and down-regulated genes in mDA and SER-neurons: **f)** Venn diagram of genes commonly and uniquely up-regulated in mDA and SER-neurons compared to NPCs. **g)** Venn diagram of genes commonly and uniquely down-regulated in mDA and SER-neurons compared to NPCs.

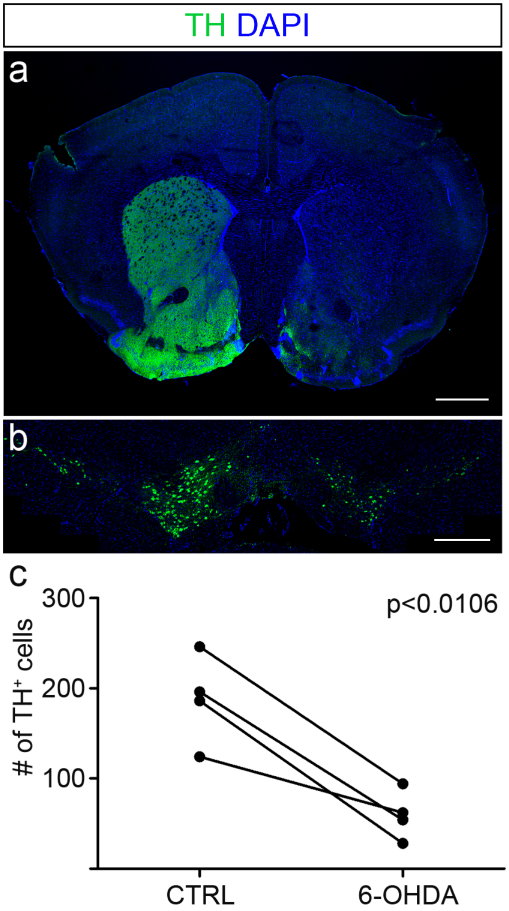
TH DAPI
a
b
c
p<0.0106
# of TH⁺ cells
300
200
100
CTRL
6-OHDA

**Supplementary Figure 10**

**a)** Immunostaining for TH (green) in the striatum shows loss of TH-immunoreactivity in the 6-OHDA injected hemisphere (right). Scale bar: 1 mm. **b)** Immunostaining for TH (green) in the ventral midbrain shows loss of TH-immunoreactivity in the 6-OHDA injected hemisphere (right). Scale bar: 500 µm. **c)** Quantification of $TH^+$ cells (green) in the ventral midbrain shows significant decrease in the 6-OHDA injected hemisphere. Significance in (**c**) was determined with a two-tailed paired Student's t-test assuming equal variances (n = 4).

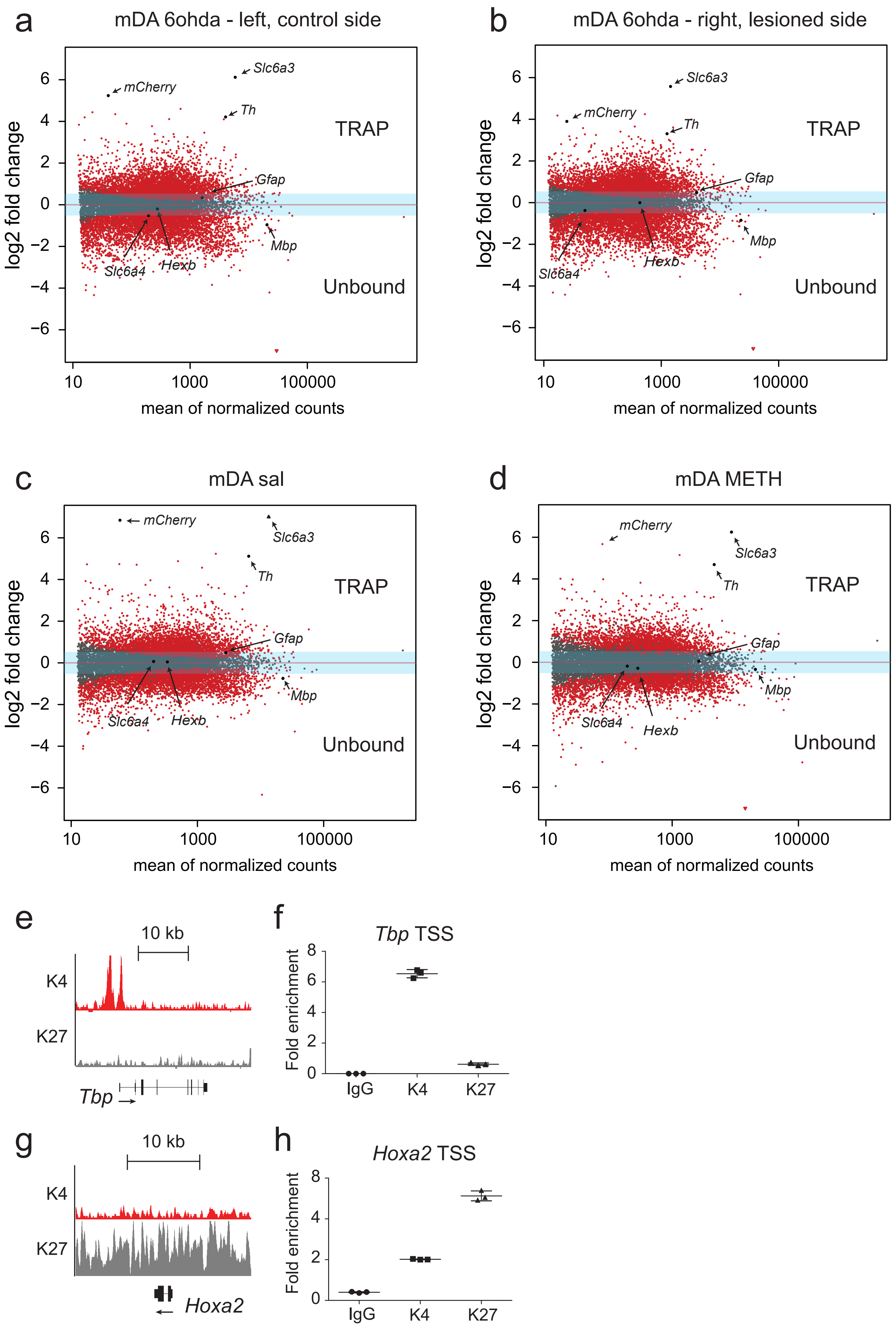
a
mDA 6ohda - left, control side
b
mDA 6ohda - right, lesioned side
c
mDA sal
d
mDA METH
log2 fold change
mean of normalized counts
TRAP
Unbound
mCherry
Slc6a3
Th
Gfap
Mbp
Slc6a4
Hexb
e
10 kb
K4
K27
Tbp
f
Tbp TSS
Fold enrichment
IgG
K4
K27
g
10 kb
Hoxa2
h
Hoxa2 TSS

**Supplementary Figure 11**

Validation of TRAP-seq efficiency: **a)** MA-analysis showing differentially expressed genes between the TRAP-fractions and the corresponding unbound fractions in the left, unlesioned control hemisphere of 6-OHDA lesioned mice (n = 5). Genes that did not change expression significantly (adj. p-value ≤0.05, Wald test) are colored in grey. The light blue area marks genes with a log2 fold change less than 0.5. **b)** As in (**a**) for differentially expressed genes between the TRAP-fractions and the corresponding unbound fractions in the right, 6-OHDA lesioned hemisphere of 6-OHDA lesioned mice (n = 5). **c-d)** As in (**a**) for TRAP-fractions and the corresponding unbound fractions of saline (n = 4) (**c**) and methamphetamine treated (n = 4) (**d**) mice, respectively. **e)** Genome browser except showing H3K4me3 (K4, red, vertical scale 0-160 RPKM) and H3K27me3 (K27, gray, vertical scale 0-100 RPKM) enrichment at the *Tbp* locus in naïve mDA nuclei. This locus serves as positive control for K4 and negative control for K27 enrichment in **Figure 6. f)** ChIP-qPCR in mDA nuclei showing fold-enrichment of IgG, K4 and K27 relative to input near the *Tbp* TSS, confirming that there was high enrichment of K4 and low enrichment of K27, relative to input (n = 3 technical replicates, bars indicate average enrichment ± SD). **g)** Genome browser except showing K4 and K27 ChIP-enrichment at the *Hoxa2* locus in naïve mDA nuclei as in (**e**). This locus serves as negative control for K4 and positive control for K27 ChIP-enrichment in **Figure 6. h)** ChIP-qPCR in mDA nuclei showing fold-enrichment of IgG, K4 and K27 relative to input near the *Hoxa2* TSS, confirming that there was high enrichment of K27 and low enrichment of K27, relative to input (n = 3 technical replicates, bars indicate average enrichment ± SD).

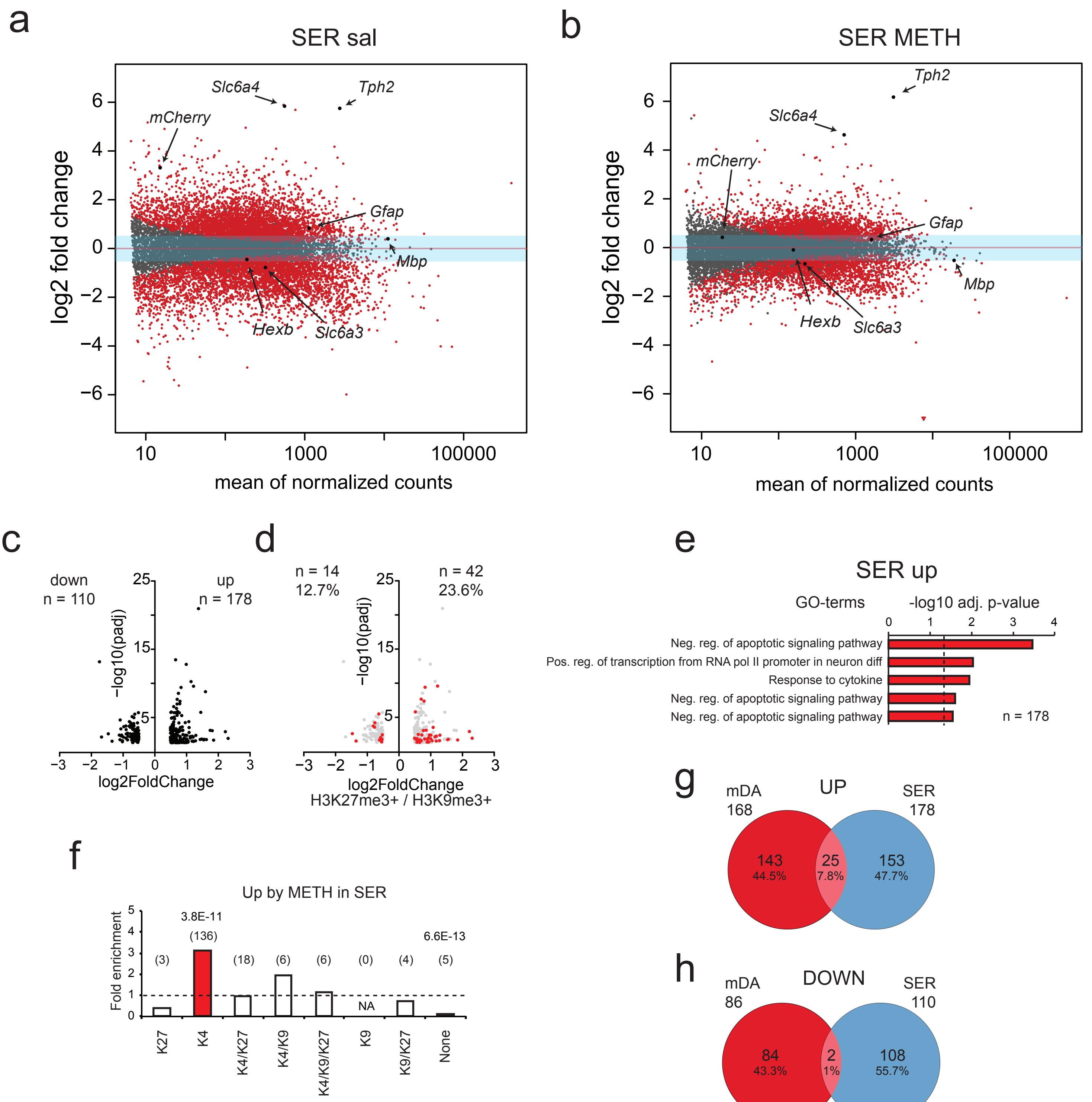
a
SER sal
log2 fold change
mean of normalized counts
Slc6a4
Tph2
mCherry
Gfap
Mbp
Hexb
Slc6a3
b
SER METH
log2 fold change
mean of normalized counts
Tph2
Slc6a4
mCherry
Gfap
Mbp
Hexb
Slc6a3
c
down
n = 110
up
n = 178
−log10(padj)
log2FoldChange
d
n = 14
12.7%
n = 42
23.6%
−log10(padj)
log2FoldChange
H3K27me3+ / H3K9me3+
e
SER up
GO-terms
-log10 adj. p-value
Neg. reg. of apoptotic signaling pathway
Pos. reg. of transcription from RNA pol II promoter in neuron diff
Response to cytokine
Neg. reg. of apoptotic signaling pathway
Neg. reg. of apoptotic signaling pathway
n = 178
f
Up by METH in SER
Fold enrichment
3.8E-11
(136)
6.6E-13
(3)
(18)
(6)
(6)
(0)
(4)
(5)
NA
K27
K4
K4/K27
K4/K9
K4/K9/K27
K9
K9/K27
None
g
mDA
168
UP
SER
178
143
44.5%
25
7.8%
153
47.7%
h
mDA
86
DOWN
SER
110
84
43.3%
2
1%
108
55.7%

**Supplementary Figure 12**

TRAP-seq after saline or methamphetamine injection in *SERT-cre-Rpl10a-mCherry* mice: a-b) MA-analysis showing differentially expressed genes between the TRAP-fractions and the corresponding unbound fractions from **a)** saline injected (n = 4), or **b)** methamphetamine injected mice (n = 4). **c)** Volcano plot showing differential gene expression in hindbrain SER-neurons after saline or methamphetamine injections. Genes that were not enriched in TRAP fraction and differentially expressed in unbound fraction were removed. Genes with an adjusted p-value of $< 0.05$ (Wald test) and a log2 fold change of $> 0.5$ are shown. **d)** As in (**a**) where H3K27me3 and/or H3K9me3-marked and regulated genes are marked with red. **e)** Gene Ontology (GO) of selected (-log10(adj. p-value), Fisher's exact test) biological processes for up-regulated genes in SER-neurons after methamphetamine injection (n = 178). **f)** Bars indicate the odds-ratio enrichment for up-regulated genes after methamphetamine injection per chromatin state. Absolute numbers of genes per state are given in parenthesis. Red bars indicate significant enrichment according to Fisher's exact test and adjusted for multiple testing. **g-h)** Venn diagrams showing the overlap between **g)** up-regulated genes in mDA-neurons after methamphetamine injection compared to SER-neurons and **h)** down-regulated genes in mDA-neurons after methamphetamine injection compared to SER-neurons.

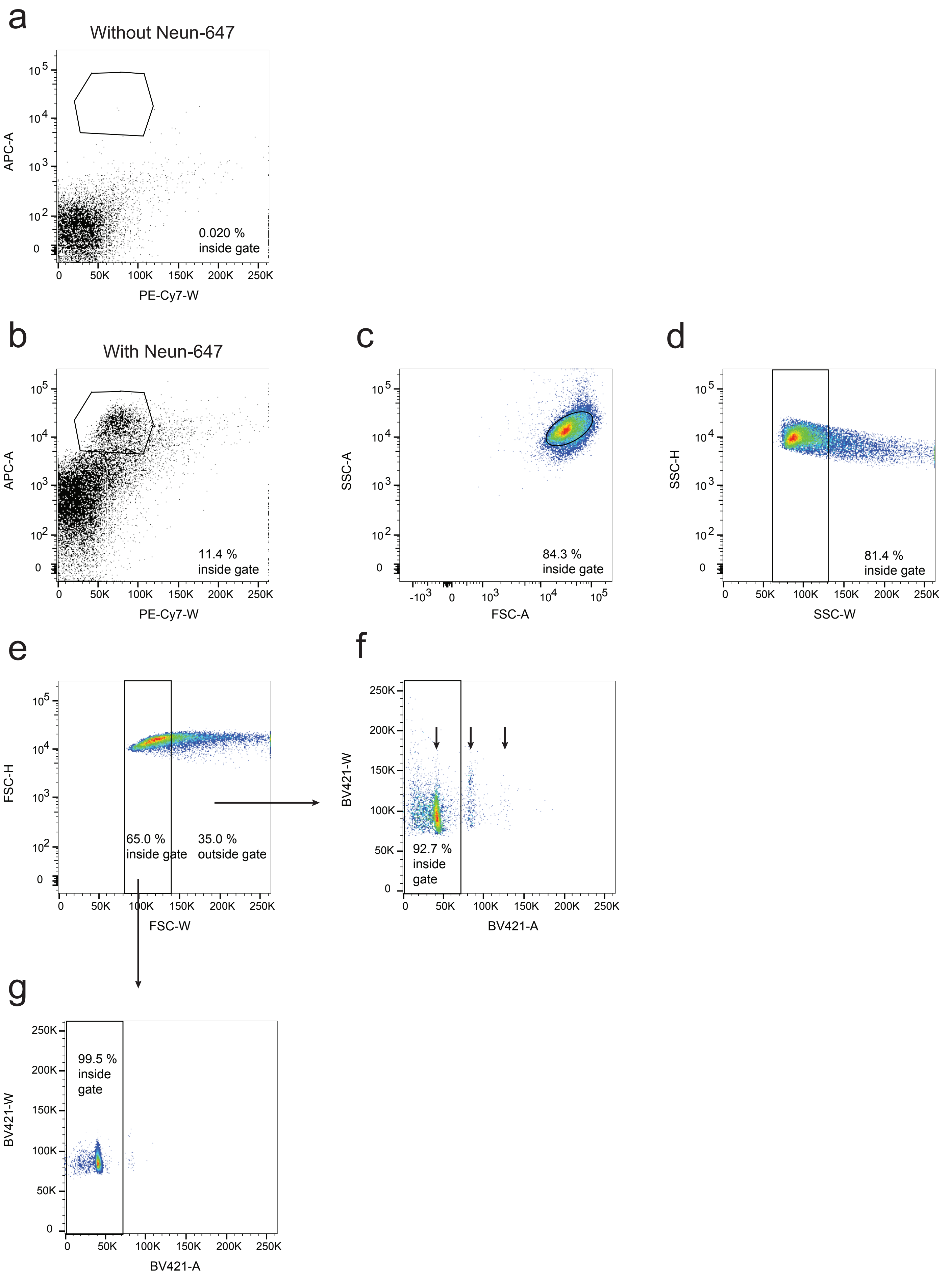
a
Without Neun-647
APC-A
PE-Cy7-W
0.020 %
inside gate
b
With Neun-647
APC-A
PE-Cy7-W
11.4 %
inside gate
c
SSC-A
FSC-A
84.3 %
inside gate
d
SSC-H
SSC-W
81.4 %
inside gate
e
FSC-H
FSC-W
65.0 %
inside gate
35.0 %
outside gate
f
BV421-W
BV421-A
92.7 %
inside
gate
g
BV421-W
BV421-A
99.5 %
inside
gate

**Supplementary Figure 13**

To examine the prevalence of multiple nuclei being detected as a single event in the FACS procedure (e.g. doublets, triplets), we turned to analyze neuronal nuclei from adult mouse cortex using an Alexa 647 labeled antibody against the pan-neuronal nuclear marker NeuN. This strategy enabled us to analyze far more sorting events than what is possible for mDA or SER-neurons. Using a similar gating strategy as for sorting mDA, SER and NPC-nuclei we found that **a-b)** NeuN positive nuclei were distinguishable from NeuN negative nuclei and that the downstream gating strategy given in **c-e)** was efficiently selective for singlet nuclei as: **f)** events falling outside the gate in **(e)** displayed multiple, distinct populations based on DAPI fluorescence (**f, arrows**) with 92.7% corresponding to singlet nuclei, whereas **g)** events falling inside the gate in **(e)** corresponded to 99.5% singlet nuclei.

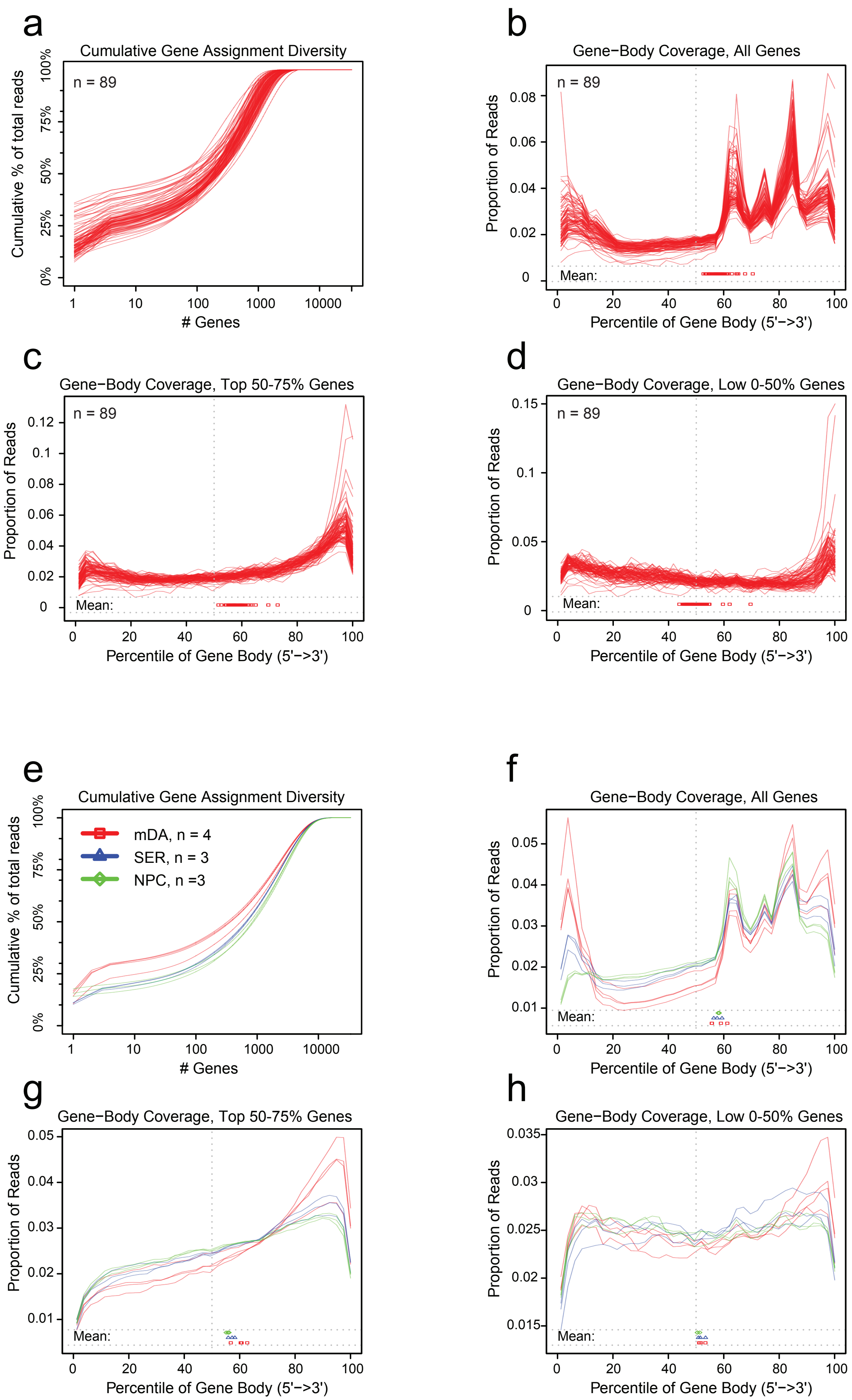

a
Cumulative Gene Assignment Diversity
n = 89
Cumulative % of total reads
# Genes
b
Gene−Body Coverage, All Genes
n = 89
Proportion of Reads
Mean:
Percentile of Gene Body (5'−>3')
c
Gene−Body Coverage, Top 50-75% Genes
n = 89
Proportion of Reads
Mean:
Percentile of Gene Body (5'−>3')
d
Gene−Body Coverage, Low 0-50% Genes
n = 89
Proportion of Reads
Mean:
Percentile of Gene Body (5'−>3')
e
Cumulative Gene Assignment Diversity
mDA, n = 4
SER, n = 3
NPC, n =3
Cumulative % of total reads
# Genes
f
Gene−Body Coverage, All Genes
Proportion of Reads
Mean:
Percentile of Gene Body (5'−>3')
g
Gene−Body Coverage, Top 50-75% Genes
Proportion of Reads
Mean:
Percentile of Gene Body (5'−>3')
h
Gene−Body Coverage, Low 0-50% Genes
Proportion of Reads
Mean:
Percentile of Gene Body (5'−>3')

**Supplementary Figure 14**

Additional quality control for RNA-seq libraries generated by the Smart-seq2 protocol. **a-d)** Analysis of the 89 single nuclei libraries that passed the QC-step according to the simpleSingleCell workflow (**Methods**). **a)** Cumulative gene assignment diversity: The top 100 expressed genes account for 25-50% and the top 1000 expressed genes account for about 75% of total reads mapping to single unique genes. **b)** Gene-body coverage profiles for 89 single nuclei libraries across quantiles of all genes' lengths, from 5' to 3'. To evaluate the putative influence of a limited number of high-coverage genes on the gene-body coverage profiles and potentially sequence-specific biases on such transcripts we also generated: **c)** gene-body coverage profiles for only the genes that are in the upper quartile by read-count (50-75%) and **d)** gene-body coverage profiles for genes in the two lower quartiles by read-count (0-50%). A clear 3'-bias can be observed uniformly in **c-d**, which is expected in libraries obtained by poly-A selection. **e-h)** Analysis of libraries obtained from 1000-nuclei batches of sorted mDA (red lines, n = 4), SER (blue lines, n = 3) and NPC (green, n = 3) nuclei. **e)** Cumulative gene assignment diversity for 4 mDA, 3 SER and 3 NPC libraries. **f)** Gene-body coverage profiles across all genes' length, from 5' to 3'. **G)** Gene-body coverage profiles for only the genes that are in the upper quartile by read-count (50-75%) and **h)** gene-body coverage profiles for genes in the two lower quartiles by read-count (0-50%). An uniform 3'-bias similar as for the single nuclei was observed as expected.

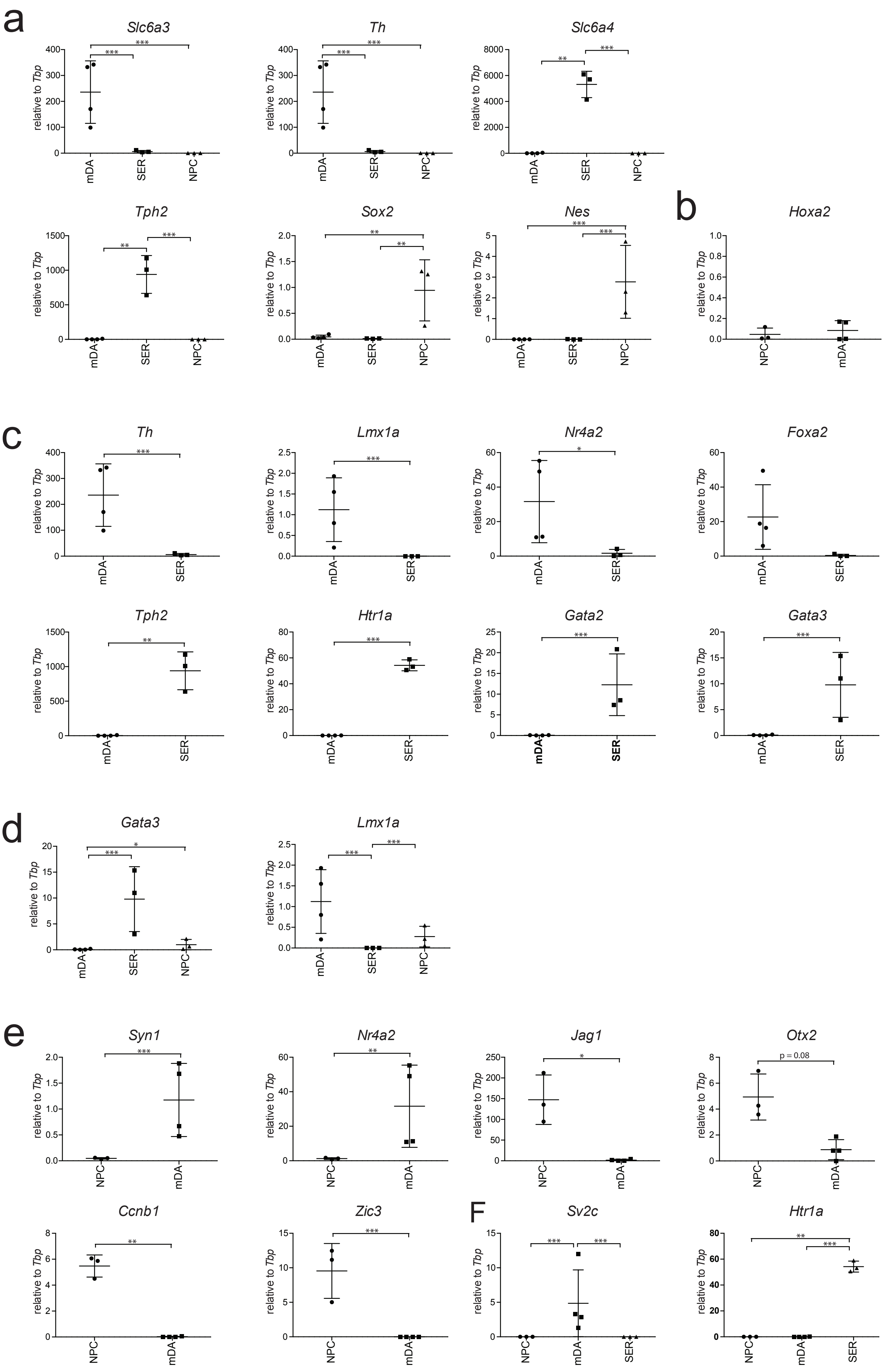
a
Slc6a3
Th
Slc6a4
Tph2
Sox2
Nes
b
Hoxa2
c
Th
Lmx1a
Nr4a2
Foxa2
Tph2
Htr1a
Gata2
Gata3
d
Gata3
Lmx1a
e
Syn1
Nr4a2
Jag1
Otx2
p = 0.08
Ccnb1
Zic3
F
Sv2c
Htr1a
relative to Tbp
mDA
SER
NPC

**Supplementary Figure 15**

Validation of gene expression levels given as RPKM in RNA-seq examples in figures by qPCR. Bars indicate average levels of the examined gene product ± SD, relative to *Tbp*. **a)** Validation of gene expression levels for representative genes given in **Fig 1**. **b)** Validation of gene expression levels for representative genes given in **Fig 4. c)** Validation of gene expression levels for representative genes given in **Fig 5. d)** Validation of gene expression levels for representative genes given in **Fig S1**. **e)** Validation of gene expression levels for representative genes given in **Fig S7**. **f)** Validation of gene expression levels for representative genes given in **Fig S9.** Significance was calculated from ddCt values according to two-tailed Student´s t-test assuming equal variances. *** $p \leq 0.001$, ** $p \leq 0.01$, * $p \leq 0.05$.

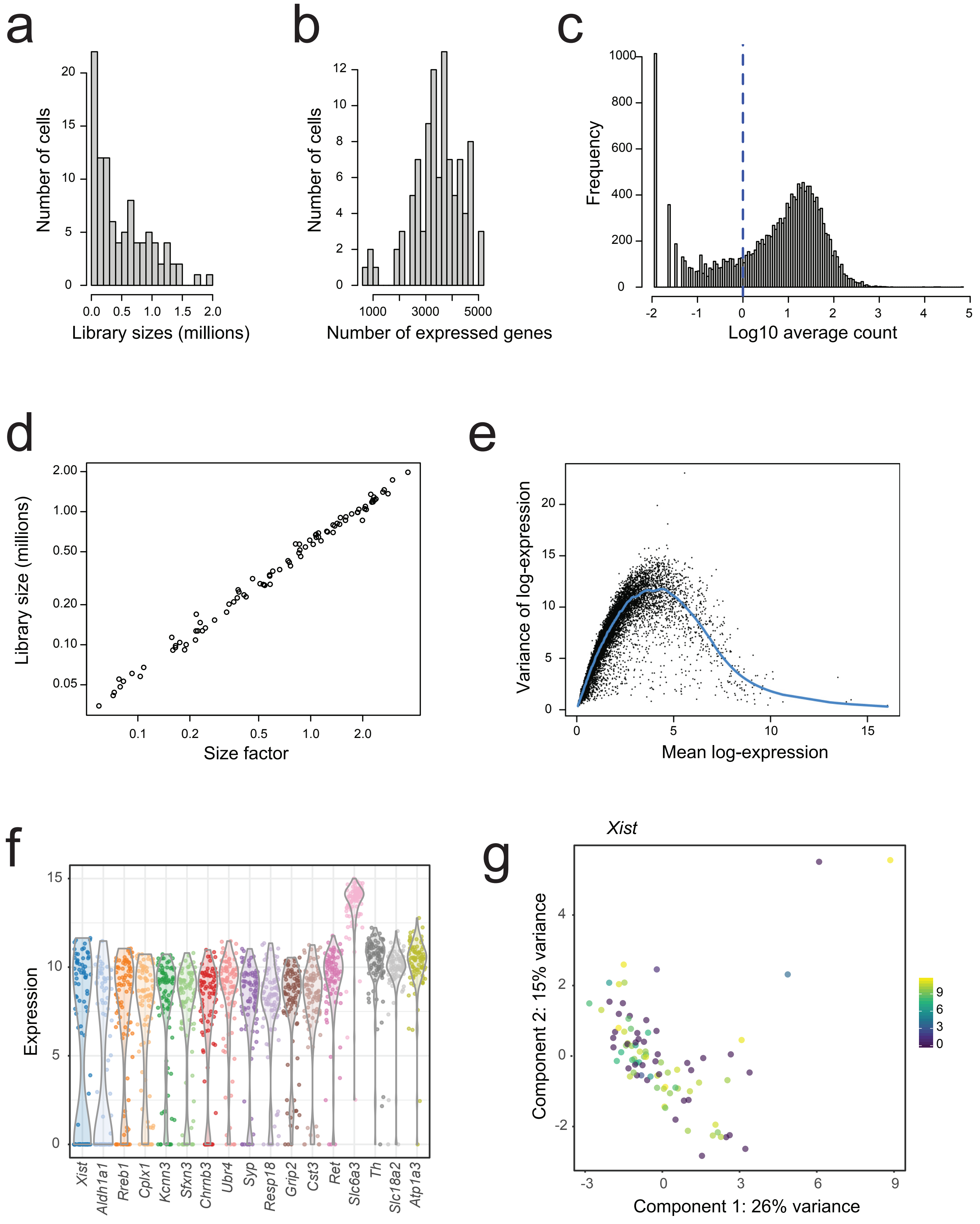
a
Number of cells
Library sizes (millions)
b
Number of cells
Number of expressed genes
c
Frequency
Log10 average count
d
Library size (millions)
Size factor
e
Variance of log-expression
Mean log-expression
f
Expression
Xist
Aldh1a1
Rreb1
Cplx1
Kcnn3
Sfxn3
Chrnb3
Ubr4
Syp
Resp18
Grip2
Cst3
Ret
Slc6a3
Th
Slc18a2
Atp1a3
g
Xist
Component 2: 15% variance
Component 1: 26% variance

**Supplementary Figure 16**

Single nuclei RNA-seq analysis quality control following the simpleSingleCell workflow. **a)** Histogram showing library sizes of all analyzed nuclei. **b)** Histogram showing number of expressed genes in all libraries. **c)** Histogram of log-average counts for all genes in the analyzed nuclei. The filter threshold is represented by the dashed line. **d)** Size factors from deconvolution, plotted against library sizes for all nuclei. Axes are shown on a log-scale. **e)** Variance of normalized log-expression values for each gene in the analyzed nuclei, plotted against the mean log-expression. The blue line represents the mean-dependent trend fitted to the variances of the endogenous genes. **f)** Violin plots of normalized log-expression values for the HVGs. Each point represents the log-expression value in a single nucleus. **g)** PCA plot constructed from normalized log-expression values of correlated HVGs, where each point represents a nucleus. First and second components are shown, along with the percentage of variance. Each cell is colored according to the expression of the *Xist* gene.

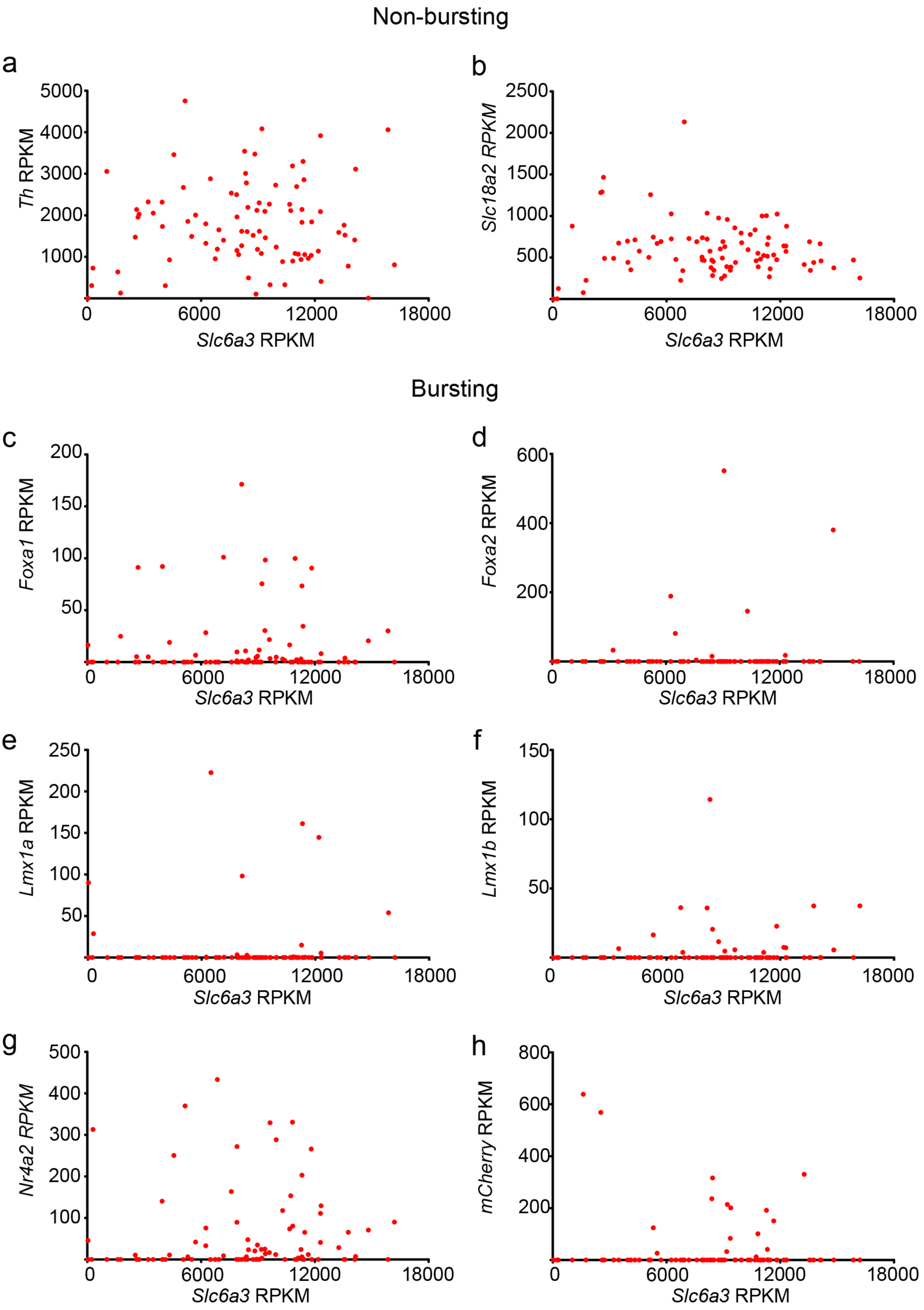

Non-bursting
a
Th RPKM
5000
4000
3000
2000
1000
0
6000
12000
18000
Slc6a3 RPKM
b
Slc18a2 RPKM
2500
2000
1500
1000
500
0
6000
12000
18000
Slc6a3 RPKM
Bursting
c
Foxa1 RPKM
200
150
100
50
0
6000
12000
18000
Slc6a3 RPKM
d
Foxa2 RPKM
600
400
200
0
6000
12000
18000
Slc6a3 RPKM
e
Lmx1a RPKM
250
200
150
100
50
0
6000
12000
18000
Slc6a3 RPKM
f
Lmx1b RPKM
150
100
50
0
6000
12000
18000
Slc6a3 RPKM
g
Nr4a2 RPKM
500
400
300
200
100
0
6000
12000
18000
Slc6a3 RPKM
h
mCherry RPKM
800
600
400
200
0
6000
12000
18000
Slc6a3 RPKM

**Supplementary Figure 17**

**a-b)** Neither *Th* (**a**) nor *Slc18a2* (**b**) exhibit bursting as determined by comparing the expression levels in single nuclei to that of *Slc6a3* (*DAT*). **c-h)** The mDA specific transcripts *Foxa1* (**c**), *Foxa2* (**d**), *Lmx1a* (**e**), *Lmx1b* (**f**), *Nr4a2* (*Nurr1*) (**g**) as well as the *DAT*-regulated *mCherry* transcript (**h**) all exhibit transcriptional bursting determined by comparing their expression levels in single nuclei to that of *Slc6a3* (*DAT*).

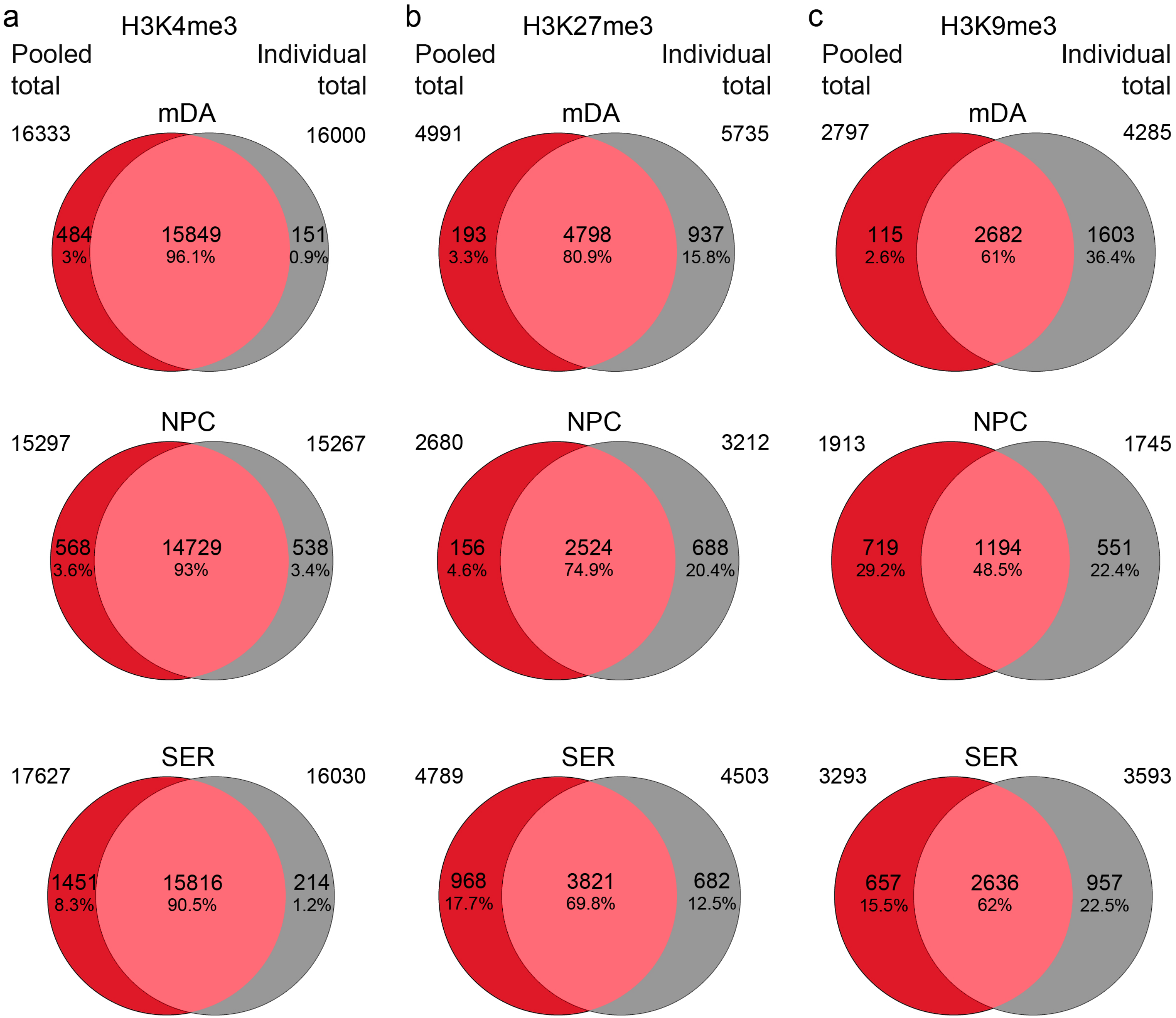
a
H3K4me3
Pooled total
Individual total
mDA
16333
16000
484
3%
15849
96.1%
151
0.9%
NPC
15297
15267
568
3.6%
14729
93%
538
3.4%
SER
17627
16030
1451
8.3%
15816
90.5%
214
1.2%
b
H3K27me3
Pooled total
Individual total
mDA
4991
5735
193
3.3%
4798
80.9%
937
15.8%
NPC
2680
3212
156
4.6%
2524
74.9%
688
20.4%
SER
4789
4503
968
17.7%
3821
69.8%
682
12.5%
c
H3K9me3
Pooled total
Individual total
mDA
2797
4285
115
2.6%
2682
61%
1603
36.4%
NPC
1913
1745
719
29.2%
1194
48.5%
551
22.4%
SER
3293
3593
657
15.5%
2636
62%
957
22.5%

**Supplementary Figure 18**

**a)** In all three cell-types, there is a comparable amount of detected genes associated with H3K4me3 between pooled and individual computation. **b)** In all three cell-types, there is a comparable amount of detected genes associated with H3K27me3 between pooled and individual computation. **c)** In all three cell-types, there is a comparable amount of detected genes associated with H3K9me3 between pooled and individual computation.
